# Some of the effective factors in the pathogenesis of gastro‐oesophageal reflux disease

**DOI:** 10.1111/jcmm.13939

**Published:** 2018-10-15

**Authors:** Fatemeh Nejat Pish‐Kenari, Durdi Qujeq, Hossein Maghsoudi

**Affiliations:** ^1^ Student Research Committee Babol University of Medical Sciences Babol Iran; ^2^ Department of Clinical Biochemistry School of Medicine Babol University of Medical Sciences Babol Iran; ^3^ Cellular and Molecular Biology Research Center (CMBRC) Health Research Institute Babol University of Medical Sciences Babol Iran; ^4^ Dental Materials Research Center Institute of Health Babol University of Medical Sciences Babol Iran; ^5^ Cancer Research Center Health Research Institute Babol University of Medical Sciences Babol Iran

**Keywords:** COX, cytokines, oesophagitis, GERD, HIF, inflammatory factors, MPO

## Abstract

Oesophageal adenocarcinoma is one of the most fatal tumours to affect the digestive tract and is the eighth most common malignancy worldwide. Gastro‐oesophageal reflux has an important role in the incidence of adenocarcinoma of the oesophagus. Gastro‐oesophageal reflux disease (GERD) is a multifactorial, acid‐peptic disorder that results from the reflux of noxious material from the stomach into the oesophagus. The refluxed material causes the occurrence of oesophageal inflammation which creates a condition that is called reflux oesophagitis. The prevalence of this disease has increased dramatically in recent decades, mostly in the western world, where it affects about 10% to 30% of the population. The aetiology of oesophageal mucosal damage is complicated. Many inflammatory mediators are produced within the gastrointestinal (GI) tract, but their contributions in pathophysiology and disease pathogenesis have not been well investigated. Despite the protective barrier provided by the oesophageal mucosa, refluxed materials can cause oxidative injury and in?ammatory responses that involve the epithelium and immune cells. The analysing cellular events in gastro‐ oesophageal reflux disease and physiological responses to such conditions are important and necessary for a better grasp of the pathogenesis of GERD and the expansion of new treatments. Therefore, we want to discuss some of the important and key factors of GERD disease in this article.

## 1 INTRODUCTION

Gastro‐oesophageal reflux disease (GERD) is widely considered a serious cause of oesophageal squamous epithelium inflammation. GERD develops due to the prolonged exposure of oesophageal cells to the acidic contents of the stomach.^1^ Nonerosive reflux disease (NERD) and erosive oesophagitis (EE) are the most common phenotypic presentations of GERD. EE is determined as the presence of a specific wound in the oesophagus of patients with or without GERD symptoms; however, patients with NERD have typical GERD symptoms and do not exhibit macroscopic lesions in the mucous membrane by endoscopy despite the mucous membranes not being normal.^1^ It should be known if oesophagitis persists, it can motive hyperplasia and Barrett's oesophagus (BE), a premalignant situation in which the normal stratified lining of the oesophagus is replaced with metaplastic specialized intestinal‐type epithelium with goblet cells.^2^ The aetiology of oesophageal mucosal damage is complicated. Due to the importance of GERD and the impact of inflammatory factors on the onset and spread of this disease, we will discuss the mechanisms that affect the development of GERD.

## 2 PATHOGENESIS OF OESOPHAGUS INFLAMMATION

For many years, it has been believed that EE results from the acid burn and chemical injury. It was thought that EE presented itself when the squamous epithelium of the oesophagus is exposed to refluxed gastric juice contents such as acid and pepsin. These substances damage the junctional complexes that hold cells together and cause the epithelium to become permeable, allowing the acid to enter and attack the epithelial cells, damage the oesophageal tissue, and cause cell death.^3^ However, unlike in the past, some researchers have reported that the complications of reflux are due to the involvement of the immune system and inflammation. In fact, acid and pepsin can induce significant macroscopic damage in the squamous cells of the oesophagus. The damaged and inflamed tissue releases inflammatory mediators detected by the immune system. Additionally, in response to these chemicals, endothelial cells produce adhesion molecules that absorb and activate leucocytes, thus causing inflammation conditions.^4^

## 3 ROS AND RNS

The reactive oxygen species (ROS) are produced by the metabolism of cells. During times of environmental stress, the ROS amount can increase significantly. This may cause notable damage to cell structures. Harmful effects of ROS are most often damage to DNA or RNA; oxidations of polyunsaturated fatty acids in lipids; oxidations of amino acids in proteins; oxidative deactivation of specific enzymes by oxidation of cofactors. Exogenous agents like UV radiation, cigarette smoking, alcohol consumption, and ingestion of nonsteroidal anti‐inflammatory drugs (NSAIDs) can cause ROS production. Moreover, the levels of ROS increase as a result of infections, ischaemia‐reperfusion (I/R) injury, and various inflammatory processes.^5^

Reactive nitrogen species (RNS) includes nitric oxide, nitrogen dioxide, and nonradical compounds, for example, peroxynitrite and dinitrogen trioxide. These free radicals are unstable owing to the presence of unpaired electrons. The RNS along with ROS can act to damage cells, causing nitrosative stress. They are involved in abroad range of diseases like ageing, I/R injury, hypertension, atherosclerosis, diabetic neuropathies, renal diseases, GERD, IBD, and cancers.^6^

## 4 COX

**Figure 1:**
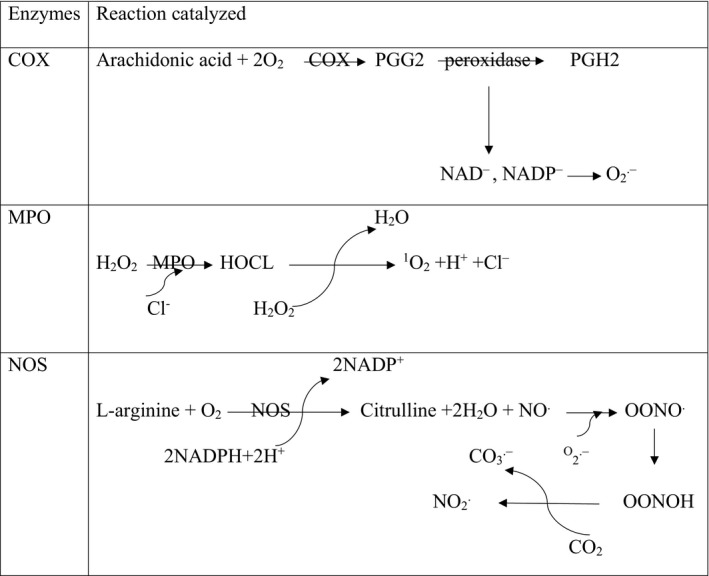
Oxidative enzymatic reactions

Some prostanoids are important biomolecules involved in inflammation. The limiting step in the formation of prostanoids is the conversion of arachidonic acid to prostaglandin H, which is carried out by the cyclooxygenase enzyme(COX). The pharmacological inhibition of COX can eliminate allergic, inflammatory, and pain symptoms.^7^ The function of NSAIDs is to inhibit COX. The COX has been shown to be expressed in at least two different isoforms: the constitutively expressed form that exists in all tissues called COX‐1, and a COX‐2 form induced by various agents, including mitogens, hormones, and cytokines. COX‐1 is a “housekeeping” gene that is expressed in most normal tissues that are important for many functions. In contrast, COX‐2 is a product of the “immediate‐early” gene; it is rapidly induced by cytokines, inflammatory mediators, endotoxin, and growth factors.^8^ There is great evidence that COX‐2 induction plays an important role in cancer progression by promoting cell proliferation, decreasing apoptosis rate, and stimulating angiogenesis. The expression of COX‐2 has often been accompanied by precancerous changes in the GI mucosa such as Barrett's oesophagus, and it is also involved in the progression of cancers. As shown in Figure 1, with the peroxidase action in PGs production pathway, NAD^−^ and NADP^−^ radicals are produced. These radicals can produce O_2_^−.^that subsequently causes the occurrence of oxidative stress.^9^

## 5 MPO

The myeloperoxidase enzyme plays a very important role in inflammatory processes and oxidative stress. This enzyme, by catalysing the respiratory burst reaction with the help of hydrogen peroxide, produces many oxidizing intermediates that cause oxidative damage to cells and tissues. This enzyme chlorinates H_2_O_2_ and converts it into highly reactive HOCl. As shown in Figure 1 during reactions MPO, O_2_ is produced, which although not a free radical, is analogous to ROS because of its electron structure. Research has shown that the MPO activity is enhanced in the inflamed mucosa of patients with ulcerative colitis disease and this may encourage the development of malignancy related to this disease.^10^

## 6 NOS

Nitric oxide synthase (NOS) produces nitric oxide (NO) from L‐arginine on the pathway for the production of L‐citrulline. This enzyme consists of three major isoforms, including constitutively expressed neuronal NOS, endothelial NOS, and cytotoxic‐inducible NOS. The iNOS mainly produce NO in response to inflammatory stimuli. The pro‐inflammatory cytokines cause iNOS expression in many cells. The excessive production of NO as an inflammatory interface can cause tissue destruction in inflammatory autoimmune disease.^11^ Depending on the amount of NO concentration, it can act as either a pro‐inflammatory or anti‐inflammatory agent. Soluble guanylyl cyclase is the main receptor of NO in the cell membrane. When NO is coupled with the soluble guanylyl cyclase, the intracellular catalytic unit is activated and catalyses the conversion of GTP to cGMP, then the protein kinase G and the kinase regulated by the external signal are activated. Activating this pathway is a mark for immigration of a cancerous cell, and it is sometimes necessary to attack tumour cells and metastasis.^12^ As shown in Figure 1, nitric oxide is a poor oxidant that can react with superoxide and form secondary power intermediates such as proxy nitrite and nitrogen dioxide, which interfere with their cytotoxic effects.^13^

## 7 HIF

**Figure 2:**
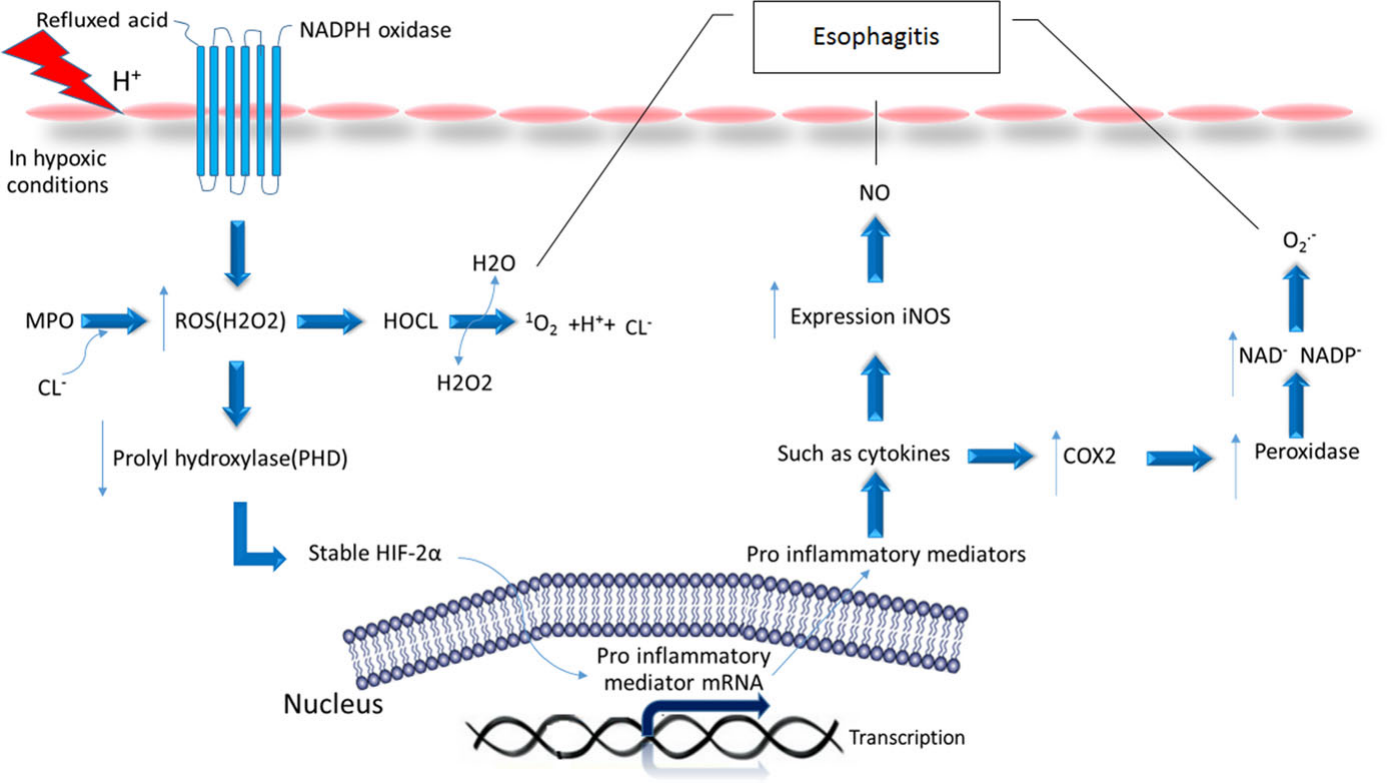
Effect of each factor in the development of oesophagitis

Inflamed tissues, such as in reflux oesophagitis, are frequently hypoxic. The hypoxia stimulates the expression of hypoxia‐inducible factors (HIFs). HIFs act in the cellular environment in response to the hypoxia during inflammatory processes. HIFs are composed of two subunits α and β. In humans, there are three different isomers of HIF‐α containing HIF‐1α, HIF‐2α, and HIF‐3α.^14^ Under normal conditions, HIFs are inactive because proteasome degrades HIF‐α subunits rapidly. This degradation begins when prolyl hydroxylases (PHDs) catalyse the hydroxylation of proline residues in the oxygen‐dependent degradation domain (ODD) of HIF‐α. Hydroxylated HIF‐α is bound by von Hippel‐Lindau (VHL) protein, which initiates degradation via the ubiquitin‐proteasome pathway. As shown in Figure 2 in hypoxic conditions, the activity of prolyl hydroxylases decreases, thus preventing the destruction of HIFs by the proteasome. This allows the HIFs to enter the nucleus by inducing transcription of target genes. In recent studies of reflux disease, it has been shown that the presence of oesophageal epithelial cells exposed to acid and bile salts triggers the activation of the NADPH Oxidase enzyme, which increases the production of ROS. Similar to hypoxic conditions, ROS also stabilizes the HIFs protein. ROS reduces the activity of prolyl hydroxylases enzyme and consequently affects the stability of HIF‐2α. Moreover, the molecular mechanisms elucidated in oesophageal squamous cell lines show that HIF‐2α activates the transcription factor NF‐kB and promotes the expression and secretion of pro‐inflammatory molecules including chemokines that attract T lymphocytes.^15^

## 8 CONCLUSION

According to the above research done, the inflammatory mediator profile in GERD indicates an inflammatory response with increased levels of inflammatory factors including ROS, RNS, COX, NOS, MPO, and HIF in the oesophageal mucosa. These data are supported by the increased activity of NF‐KB in the mucosa of GERD patients. NF‐KB can be activated by pro‐inflammatory cytokines and further facilitates the inflammatory mediator synthesis and secretion. Therefore, by increasing these factors, the damage to the oesophagus increases, which, if continued, can progress to oesophageal cancer. Therefore, they have an important role in the pathogenesis of GERD.

## Conflicts of interest

The authors confirm that there are no conflicts of interest.

## References

[1] Fass R . Erosive esophagitis and nonerosive reflux disease (NERD): comparison of epidemiologic, physiologic, and therapeutic characteristics. J Clin Gastroenterol. 2007;41(2):131‐137. 17245209 10.1097/01.mcg.0000225631.07039.6d

[2] Spechler SJ , Fitzgerald RC , Prasad GA , Wang KK . History, molecular mechanisms, and endoscopic treatment of Barrett's esophagus. Gastroenterology. 2010;138(3):854‐869. 20080098 10.1053/j.gastro.2010.01.002 PMC2853870

[3] Huo X , Souza RF . Acid burn or cytokine sizzle in the pathogenesis of heartburn? J Gastroenterol Hepatol. 2013;28(3):385‐387. 23441717 10.1111/jgh.12103 PMC3589733

[4] Souza RF . From reflux esophagitis to esophageal adenocarcinoma. Dig Dis. 2016;34(5):483‐490. 27331918 10.1159/000445225 PMC4936412

[5] Bhattacharyya A , Chattopadhyay R , Mitra S , Crowe SE . Oxidative stress: an essential factor in the pathogenesis of gastrointestinal mucosal diseases. Physiol Rev. 2014;94(2):329‐354. 24692350 10.1152/physrev.00040.2012 PMC4044300

[6] Pavlick KP , Laroux FS , Fuseler J , et al. Role of reactive metabolites of oxygen and nitrogen in inflammatory bowel disease1, 2. Free Radic Biol Med. 2002;33(3):311‐322. 12126753 10.1016/s0891-5849(02)00853-5

[7] O'Neill GP , Ford‐Hutchinson AW . Expression of mRNA for cyclooxygenase‐1 and cyclooxygenase‐2 in human tissues. FEBS Lett. 1993;330(2):157‐160. 10.1016/0014-5793(93)80263-t 8365485

[8] Smith WL , DeWitt DL , Garavito RM . Cyclooxygenases: structural, cellular, and molecular biology. Annu Rev Biochem. 2000;69(1):145‐182. 10966456 10.1146/annurev.biochem.69.1.145

[9] Kukreja RC , Kontos HA , Hess ML , Ellis EF . PGH synthase and lipoxygenase generate superoxide in the presence of NADH or NADPH. Circ Res. 1986;59(6):612‐619. 3028671 10.1161/01.res.59.6.612

[10] Davies MJ . Myeloperoxidase‐derived oxidation: mechanisms of biological damage and its prevention. J Clin Biochem Nutr. 2010;48(1):8‐19. 21297906 10.3164/jcbn.11-006FR PMC3022070

[11] Iijima K , Shimosegawa T . Involvement of luminal nitric oxide in the pathogenesis of the gastroesophageal reflux disease spectrum. J Gastroenterol Hepatol. 2014;29(5):898‐905. 24863184 10.1111/jgh.12548

[12] Jadeski LC , Hum KO , Chakraborty C , Lala PK . Nitric oxide promotes murine mammary tumour growth and metastasis by stimulating tumour cell migration, invasiveness and angiogenesis. Int J Cancer. 2000;86(1):30‐39. 10728591 10.1002/(sici)1097-0215(20000401)86:1<30::aid-ijc5>3.0.co;2-i

[13] Hofseth LJ , Hussain SP , Wogan GN , Harris CC . Nitric oxide in cancer and chemoprevention1. Free Radic Biol Med. 2003;34(8):955‐968. 12684081 10.1016/s0891-5849(02)01363-1

[14] Loboda A , Jozkowicz A , Dulak J . HIF‐1 and HIF‐2 transcription factors—similar but not identical. Mol Cells. 2010;29(5):435‐442. 20396958 10.1007/s10059-010-0067-2

[15] Huo X , Agoston AT , Dunbar KB , et al. Hypoxia‐inducible factor‐2α plays a role in mediating oesophagitis in GORD. Gut 2017;66(9):1542‐1554. 27694141 10.1136/gutjnl-2016-312595 PMC5464991

